# Supplementary material for: Chemerin15 inhibits neutrophil-mediated vascular inflammation and myocardial ischemia-reperfusion injury through ChemR23
Source: EMBO Rep. 2013 Sep 3;14(11):999–1007. doi: 10.1038/embor.2013.138 (PMC3818079; doi:10.1038/embor.2013.138)
Supplement: Supplementary Information [file embor2013138s1.pdf]

SUPPLEMENTARY METHODS

Ethics All animal studies were conducted with ethical approval from the Queen Mary University of London Local Ethical Review Committee and in accordance with the UK Home Office regulations (Guidance on the Operation of Animals, Scientific Procedures Act, 1986). Human cells were prepared according to a protocol approved by the East London & The City Local Research Ethics Committee (Ref. 05/Q0603/34 ELCHA, London, United Kingdom).

Chemerin and ChemR23 antagonists Chemerin protein was sourced from R&D Systems (Abingdon, UK) and prepared according to manufacturers instructions. CCX2005 is a ChemR23 antagonist provided by Chemocentryx (California) and used at 100 nM alongwith DMSO control for *in vitro* experiments. The ChemR23 antagonist CCX832 (2 mg/100 μl) was injected subcutaneously 24 h and immediately before commencing animal preparation for myocardial infarct. Plasma was harvested at the end of the procedure and samples analysed to verify appropriate levels of circulating antagonist (> 1 μM).

Calcium mobilisation assay Calcium mobilisation assays were performed as previously described [23]. Briefly, freshly prepared PMN were incubated with 2 μM Fura 2-AM (Molecular Probes, Invitrogen, Paisley, UK) in HBSS supplemented with 2 mM $CaCl_2$ at 37°C for 1 h in the dark. Subsequently, cells were washed and plated in black clear-bottom 96-well plates. Cells were treated with vehicle, C15, C15-S (both 10 pM), chemerin (1 nM), ChemR23 antagonist CCX2005 (100 nM), or ionomycin (1 μM, used as a positive control). Mobilisation of intracellular calcium was measured by recording the ratio of fluorescence emission at 510 nm after sequential excitation at 340 and 380 nm using the NOVOstar (BMG Labtech, Aylesbury, UK) microplate reader.

ChemR23 staining for flow cytometry and immunofluorescence ChemR23 antibody (R&D Systems, Abingdon, UK) and isotype control (IgG_3; R&D Systems) were titrated 0.1 μg/ml – 80 μg/ml (0.005 μg/test – 4 μg/test) and a sub-saturation dose of 10 μg/ml (0.5 μg/test) chosen. Human whole blood (50 μl) was treated with vehicle, TNFα (10 ng/ml), fMLF (1 μM), IL-8 (100 ng/ml; PeproTech, London, UK), Annexin A1 (10 nM, R&D Systems, Abingdon, UK), α melanocyte stimulating hormone (αMSH; 10 nM) or C15 (10 pM) for 30 min followed by washing. Whole blood aliquots were incubated with anti-ChemR23 or isotype control (both R&D Systems) for 1 h at 4°C, washed and stained with goat anti-mouse IgG-Alexa Fluor 488 (Invitrogen) for 30 min. In some experiments neutrophils were exposed to Human Umbilical Vein Endothelial Cells (HUVECs) under shear conditions and their expression of ChemR23 analysed.

For immunofluorescence, isolated neutrophils were allowed to adhere to ICAM-1 coated multispot slides as outlined below. Neutrophils were fixed (2% PFA), permeabilized and blocked (10% goat serum + 0.3% Triton X-100), incubated with anti-ChemR23 (10 μg/ml) or isotype control for 1 h at room temperature, then washed and stained with goat anti-mouse IgG-Alexa Fluor 488 for 30 min. Alexa 647 conjugated wheat germ agglutinin (WGA) was used as per manufacturer's instructions to stain the cell membrane (Invitrogen) in Fig 1D. In Fig 1E Anti-CD35 (secretory vesicles), anti-CD66b (specific granules) and anti-CD63 (azurophil granules) were added for 45 min to stain neutrophil granules. Fluorescence photomicrographs were taken of representative cells using a Leica SP5 laser scanning confocal microscope, fitted with a 63x oil immersion objective and UV diode, Ar/ArKr, and HeNe lasers exciting at 405nm, 488nm and 594nm respectively. Images were analysed using NIH ImageJ 1.47 software (NIH, Bethesda, MD, USA). Virtually all cells appeared as per the examples shown in Fig1.

For detection of murine ChemR23, whole blood was collected from wildtype and ChemR23$^{-/-}$ mice by cardiac puncture into EDTA and stained with anti-mChemR23 (15 μg/ml, R&D Systems) or IgG_{2A} isotype control for 1 h at 4°C followed by goat anti-rat IgG Alexa-647 (10 μg/ml, Invitrogen) for 30 min. The number of circulating neutrophils did not significantly differ between wildtype and ChemR23$^{-/-}$ mice under baseline conditions or 2 h post-TNFα challenge. However, ChemR23$^{-/-}$ mice do exhibit monopenia (0.55x10^{6} versus 1.13x10^{6} in wildtype).

Whole blood flow cytometry Fresh venous blood (anticoagulated with 3.2% sodium citrate in a 1:10 ratio) was collected from healthy non-smoking volunteers. Whole blood aliquots (50 μl) were preincubated with C15 or vehicle (PBS) for 10 min prior to stimulation with TNFα (10 ng/ml for 20 min, Invitrogen) at 37°C. Immunoflourescence labelling of cell surface antigens was then performed. Integrin activation (presence of high affinity conformation) was assessed using antibodies that specifically detect the neo-epitope exposed on activated integrins when they adopt an extended high affinity conformation. Mouse anti-human CD11b-FITC (total; Biolegend), CD62L-PE (CD62L; Biolegend), CD18 activation epitope (mAb24, a generous gift from Prof Nancy Hogg; Cancer Research UK), CD11b-FITC activation epitope (Biolegend), CD11a activation epitope (MEM-83, Abcam) and CD29 activation epitope (HUTS-21, BD Biosciences). Anti-CD11b-FITC (activation epitope; 10 μl/test), mAb24 (2 μl/test), anti-CD11a (activation epitope, 5 μg/ml) and anti-CD29 (activation epitope, 2.5 μg/ml) were added to whole blood aliquots (50 μl) at the same time as TNFα-stimulation and unconjugated antibodies detected with rabbit anti-mouse IgG-Alexa Fluor 488 (Invitrogen) for 30 min at 4°C. CD11b-FITC (total CD11b; 5 μl/test) and CD62L-PE (3

μl/test) staining was performed at 4°C for 1 h. After washing, erythrocytes were lysed using BD FACS lysis solution and cells were washed twice, resuspended in 2% PFA and analyzed using a FACSCalibur (Becton Dickinson, Oxford, UK) and FlowJo software (OR, USA). In some experiments cells were activated with PMA (1 μM) or fMLF (1 nM) following C15 pre-treatment.

Integrin clustering Neutrophils were prepared from human blood according to standard protocol (histopaque 1077 and 1119 layering in 15 ml falcon tubes). Cells were cultured in RPMI + 5% FCS + Pen/Strep supplemented with 1mM $CaCl_2$/0.5mM $MgCl_2$. Neutrophils in suspension were treated for 10 min with 10 pM C15 or vehicle followed by 10 min with 10 ng/ml TNFα. Cells were fixed for 15 min at room temperature with 2% PFA then mounted four-well chamber slides and blocked with 10% goat serum for 30 min at room temperature. Cells were then stained with 1 μg/ml anti-CD11a (clone 27, BD Biosciences) or 10 μg/ml anti-CD11b (clone ICRF44, R&D systems) in 1% goat serum for 1 h at room temperature followed by goat anti-mouse Alexa-488 conjugated IgG (Invitrogen, 1/200 in 1% goat serum) for 30 min at room temperature. Nuclei were stained with 50 ng/ml DAPI for 5 min in dH_2O. Fluorescence photomicrographs were taken of representative cells using a Zeiss LSM confocal microscope, fitted with a 63x oil immersion objective and UV diode and Ar/ArKr lasers exciting at 405nm and 488nm respectively. Images were analysed using NIH ImageJ 1.47 software (NIH, Bethesda, MD, USA). For analysis of the proportion of the population showing clustering, images were taken using an EVOS FL cell imaging system (Life Technologies, UK) fitted with a 20x objective, and the percentage of cells clearly exhibiting integrin clustering was calculated by an observer blind to treatment group.

Chemotaxis assay in IBIDI μ-slide chemotaxis chambers Directional chemotaxis of primary human neutrophils was analysed by live cell tracking using IBIDI μ-slide chemotaxis chambers according to manufacturer's instructions (IBIDI, Munich, Germany). Briefly, chemotaxis slides were coated with 2.5 μg/ml rhICAM-1 (eBioscience, Hatield, UK; in coating buffer containing 150 mM NaCl, 20 mM TrisHO 2 mM $MgCl_2$, pH 9) for 1 h, then washed thoroughly with dH_2O and dried overnight. Neutrophils ($3x10^6$/ml) were seeded into the observation channel of the chamber, and allowed to adhere for 5 mins. The chamber reservoirs were filled with RPMI (Gibco, Paisley, UK) supplemented with $CaCl_2$ (133 mg/ml) and $MgCl_2$ (200 mg/ml; both Sigma, Kent, UK) ± C15 peptide (100 pM). Chemotactic stimulus (fMLF, 2.5 μM (to generate a final gradient of 1 μM)) was added to the right-hand reservoir and the slide mounted on an HT-200 heated/humidified stage (IBIDI) on a Nikon Eclipse TE3000 Microscope (Nikon, Melville, NY) and filmed using a Q-imaging Retiga EXi digital video camera with ImagePro-Plus software (Media Cybernetics, Wokingham, UK).

After 15 mins of equilibration, time-lapse video microscopy was performed for 30 mins at x10 magnification. Subsequently, migration of 30 cells selected from a predetermined area was tracked manually with the ImageJ manual tracking plug-in, and data were analysed with the IBIDI chemotaxis and migration tool (IBIDI). Black paths depict cells with net migration leftward, and red paths depict cells with net migration rightward towards fMLF.

ICAM-1 static adhesion assay Multispot slides were coated with rhICAM-1 (2.5 μg/ml; Ebioscience) in coating buffer (150 mM NaCl, 20 mM Tris-HCl, 2 mM $MgCl_2$, pH 9.0) for 3 h at room temperature, washed (PBS) and blocked (10% BSA) for 1 h. Human neutrophils ($2.5x10^6$/ml) were pre-treated with vehicle (PBS + Ca^{2+}/Mg^{2+}) or C15 for 10 min and 80 μl placed on rhICAM-1 coated slides in the presence of TNFα (10 ng/ml) for 10 min at 37°C. Slides were then placed under an Eclipse TE3000 microscope (Nikon, Melville, NY) with 20x magnification and six randomly selected fields photographed using a JVC TK-C1360B digital colour camera for subsequent offline analysis. Neutrophil-ICAM-1 interactions were classified as attached or spread. In some experiments neutrophils were pre-treated with ChemR23 antagonist CCX2005 (100 nM) or DMSO control for 10 min prior to C15 administration.

ICAM-1-Fc binding assay Murine bone marrow leukocytes from wildtype and $ChemR23^{-/-}$ mice were harvested and resuspended in HBSS containing $MgCl_2$ 2 mM, $CaCl_2$ 1 mM, 20 mM HEPES, and 0.1 % BSA, pH 7.4. Cells (2.5 $x10^6$/ml) were pre-treated with vehicle or C15 (10 pM) for 10 min and then incubated with TNFα (10 ng/ml) and 20 µg/mL soluble rmICAM-1/Fc chimera (R&D Systems) for 1, 5, 10 or 20 min. The reaction was stopped with ice cold HBSS containing 1% PFA and immersing cells in ice. After washing, binding of sICAM-1/Fc chimera was detected on neutrophils by staining with Ly6G-Alexa 647 and FITC-conjugated goat anti–human F(ab')2 fragment to human IgG-Fc for 30 min at 4°C. Control treatments included at the 5 min time point were $MgCl_2$ (1 mM), EDTA (5 mM) and F(ab')2 fragment only. Samples were analyzed by flow cytometry.

Fibronectin adhesion assay Murine bone marrow leukocytes from wildtype and $ChemR23^{-/-}$ mice were harvested and resuspended in HBSS containing $MgCl_2$ 2 mM, $CaCl_2$ 1 mM, 20 mM HEPES, and 0.1 % BSA, pH 7.4. Cells (2.5 $x10^6$/ml) were pre-treated with vehicle or C15 (10 pM) for 10 min and allowed to adhere to multispot slides coated with fibronectin (30 μg/ml, Invitrogen) in the presence of TNFα (10 ng/ml, Peprotech) for 1, 5, 10 or 20 min. The reaction was stopped with ice cold HBSS containing 1% PFA. After washing, neutrophil adhesion to fibronectin was detected by blocking with 2.4G2 CD16/32 Fc block (BD Biosciences) and staining with Ly6G-PE (Biolegend) for 30 min at room temperature.

Fluorescent micrographs were taken using an EVOS FL cell imaging system fitted with a 20x objective and number of adherent Ly6G positive cells quantified.

Phospho-flow cytometry Whole blood aliquots (100 μl) were treated with C15 (10 pM) or vehicle control for 10 min prior to a 10 min stimulation with TNFα (10 ng/ml). Cells were fixed (3% PFA, 15 min room temperature), erythrocytes lysed (0.1% Triton X-100, 30 min room temperature) and cells permeabilized (70% ice cold methanol, 10 min on ice) and then stained for phospho proteins (60 min at room temperature). Rabbit anti-human pSyk (Y525/526; 1/300), pErk (Y202/204; 1/200) and pSrc (Y527; 1/200) were used to investigate signalling enzyme activation state (all from Cell Signal Technology, Danvies, US), with anti-rabbit Alexa-488 secondary antibody (Invitrogen, Paisley, UK). In this case, pSyk and pErk represent activated enzymes, whilst Y527 of Src is constitutively phosphorylated to maintain the enzyme in an inactive state. Thus loss of phospho Y527 signal is associated with Src activation.

Flow chamber assay HUVECs were stimulated with TNFα (10 ng/ml) for 4 h in the presence or absence of C15. The chamber was placed under an Eclipse TE3000 microscope with 20x magnification and neutrophils were pre-treated with C15 or vehicle (PBS + Ca^{2+}/Mg^{2+}) for 10 min, and perfused over endothelial monolayers for 8 min at a rate of 1 dyne/cm^2 using a syringe pump (Harvard Apparatus Inc, South Natick, MA, USA). Six randomly selected fields were recorded for 10 s each using a JVC TK-C1360B digital color video camera for subsequent offline analysis. Neutrophil-endothelial cell interactions were automatically classified during offline analysis as rolling, adherent or transmigrated, with total interactions representing the sum of all events using ImagePro Plus software (Marlow, UK). In some experiments, neutrophils were pre-treated with C15 or vehicle and perfused over endothelial monolayers for 3 min and then perfused with TNFα to activate the neutrophils. The activated neutrophil protocol resulted in elevated adhesion and transmigration with reduced rolling interactions.

In vivo methods

Intravital Microscopy (IVM) Mice (10-15 g) were administered C15 (0.3 ng/kg) i.p followed by TNFα (500 ng, Invitrogen) 30 min later. Animals were anaesthetized with xylazine (10 mg/kg) and ketamine (200 mg/kg) i.p. 0, 2 and 4 h post-TNFα administration. The mesenteric vascular bed was exteriorized, mounted on a Zeiss Axioskop FS with a water-immersion lens (magnification: x40, Zeiss) and an eyepiece (magnification: x10, Zeiss) used to observe the microcirculation. The preparation was transilluminated with a 12-V 100-W

halogen light source and superfused with 37°C bicarbonate-buffered solution (g/L: NaCl 7.71; KCL, 0.25; $MgSO_4$, 0.14; $NaHCO_3$, 1.51; and $CaCl_2$, 0.22, pH 7.4). A Hitachi charge-coupled device color camera (model KPC571) acquired images that were displayed onto a Sony Trinitron color video monitor (model PVM 1440QM) and recorded onto DVD for subsequent offline analysis. 3-6 postcapillary venules (diameter 20-40 μm) were observed per animal and recorded for offline analysis. The extent of the inflammatory response was analyzed by measuring leukocyte rolling velocities (V_{WBC}, μm/s) and quantifying neutrophil adhesion and emigration. Leukocytes were considered adherent if they remained stationary for 30 s or longer, whereas leukocyte emigration from the microcirculation into the tissue was quantified by counting the number of cells in the perivascular tissue up to 50 μm away from the vessel wall. A previous study indicated that the majority of leukocytes interacting with the activated endothelium at early 2-4 h time points were neutrophils [20]. Venule diameters and blood flow velocities were similar in knockout and wildtype mice and animals treated with C15, eliminating the possibility that changes in hemodynamics contribute to the effect of C15 or phenotype of the ChemR23$^{-/-}$ mice.

***In vivo* model of Acute Myocardial Infarct (AMI)** The ChemR23 antagonist CCX832 (2 mg/100 μl) or vehicle control were injected subcutaneously 24 h and immediately prior to commencing animal preparation for myocardial infarct. C57BL/6 mice (5-7 weeks) were anaesthetized as for IVM and injected intravenously with C15 (0.3 ng/kg) or vehicle control. A midline cervical incision separating the skin, muscle and tissue covering the trachea was performed. Subsequently a hole was made between two cartilage rings below the glottis of the trachea allowing cannulation to maintain artificial ventilation (Harvard Apparatus, United Kingdom, 687). The tidal volume of the respirator was set at 200 μl, with the rate set at 100 strokes/min. A left thoracotomy was performed and the pericardium removed to expose the heart. A silk ligature (Mersilk, 7-0) was placed around the Left Anterior Descending Coronary Artery (LADCA) for 30 minutes. 2 hours of reperfusion followed the occlusion. Mice were kept at body temperature (37°C) by a homeothermic blanket (Harvard Apparatus, United Kingdom) during all the procedures. At the end of the reperfusion heart and blood were harvested for further analysis.

Troponin I and Myeloperoxidase activity Plasma Troponin-I level was measured using a mouse cardiac troponin-1 kit according to manufactures instructions (Life Diagnostics, West Chester, PA, 2010-1-HSP). Myeloperoxidase **(**MPO) enzyme activity was assayed by measuring hydrogen peroxide dependent oxidation of *O*-dianisidine dihydrocholoride. Blood-free tissue samples were homogenised in cell lysis tubes (Peqlab, United Kingdom, 91-PCS-CK14) containing a solution of 0.5% hexadecyltrimethylammonium bromide (HTAB, pH 6,

Sigma). The homogenized tissues were centrifuged at 15000rpm for 5 min. Supernatants were added to a buffer containing distilled water, 1% hydrogen peroxide (Sigma-Aldrich, United Kingdom, 7722-84-1) and *O*-dianisidine dihydrocholoride solution (Sigma-Aldrich, United Kingdom, 20325-40-0). Optical density readings were taken at 450 nm using the NOVOstar microplate reader (BMG LABTECH Ltd., Aylesbury, United Kingdom). Results were normalized to the protein content, determined with the Pierce BCA Protein Assay Kit (Thermo Scientific, Rockford, USA, 23225).

Supplementary figure legends

Supplementary Figure 1. Flow cytometry histograms for Fig.2 A Representative flow cytometry histograms showing the effect of 20 min neutrophil treatment with vehicle, TNFα (10 ng/ml) or C15 (10 pM), on L-selectin and PSGL-1 expression. **B** Representative flow cytometry histograms showing the effect of neutrophil pre-treatment with vehicle or C15 (10 pM) for 10 min prior to 20 min TNFα on total CD11b and CD18 and CD11b activation. Representative of 4-6 donors.

Supplementary Figure 2. Lack of effect of C15 on total CD11b expression and degranulation Neutrophils were treated with vehicle or C15 (0.1-100 pM; **A**), 10 pM (**B**) followed by 20 min stimulation with TNFα or vehicle. Cells were then stained for total CD11b expression (**A**) or markers of degranulation of secretory vesicles (CD35) and specific granules (CD66b; **B**). Data are expressed as means ± SEM for n=3 donors.

Supplementary Figure 3. Effect of C15 on human neutrophil β1 integrin activation and adhesion of murine neutrophils to β1 ligand fibronectin A Human neutrophils were pre-treated with C15 (1-1000 pM) or vehicle for 10 min followed by stimulation with TNFα (10 ng/ml) for 20 min. Cells were then stained for CD29 activation with HUTS-21 which detects the activation epitope of β1 integrin. **B** Wildtype and ChemR23$^{-/-}$ bone marrow-derived leukocytes were pre-treated with C15 (10 pM) for 10 min and allowed to adhere to multispot slides coated with fibronectin (30 μg/ml) in the presence of TNFα (10 ng/ml) for 1, 5, 10 or 20 min. Adherent neutrophils were then stained with Ly6G-PE and analysed by fluorescence microscopy. Data are expressed as means ± SEM for n=4 mice per genotype or 4 human donors.

Supplementary Figure 4. Specificity of C15's effects on integrin activation. A Leukocytes were pre-treated with vehicle or C15 (10 min) followed by challenge with PMA (lymphocytes) or TNFα (monocytes) for 20 min and then staining for CD18 activation epitope. **B** Monocytes were pre-treated with vehicle or C15 (10 min) followed by challenge with TNFα (20 min) and staining for the β1 integrin (CD29) activation epitope. **C** Neutrophils were pre-treated with vehicle or C15 (10 min) followed by challenge with fMLF (20 min) and staining for the CD11b neoepitope. Data are expressed as means ± SEM for 3 independent experiments.

Supplementary Figure 5. HUVEC treatment with C15 does not significantly affect neutrophil interactions. HUVECs were treated with TNFα (10 ng/ml) and vehicle or C15 (1-100 pM) for 4 h. Isolated human neutrophils were then flowed over the HUVECs and

neutrophil-endothelial cell interactions classified as rolling, adhesion or transmigration. Data are expressed as means ± SEM for 4 independent experiments.

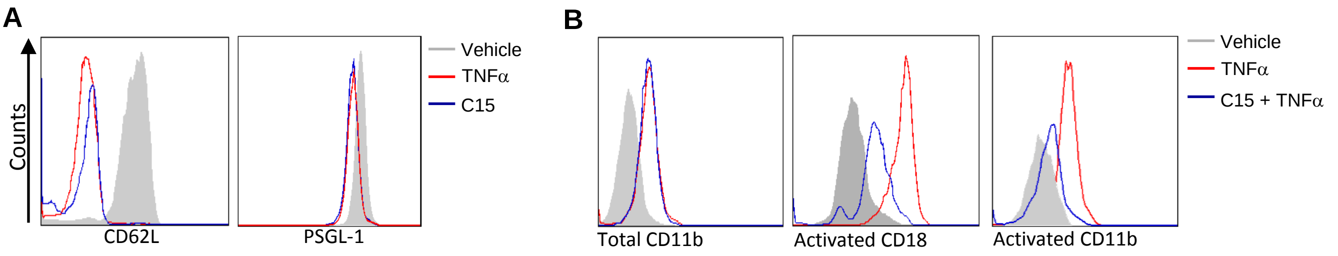

Supplementary Fig 1.
Representative flow cytometry histograms for Fig.2

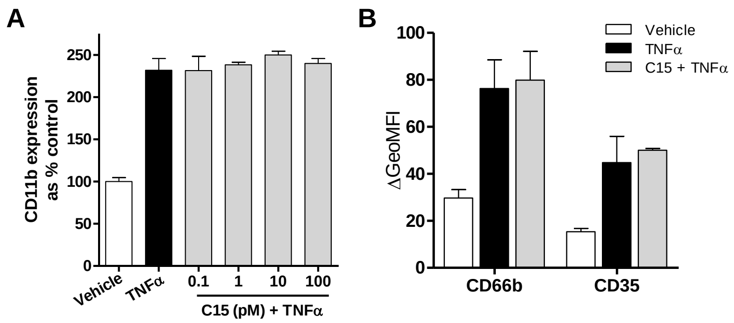

Supplementary Fig 2.
Lack of effect of C15 on total CD11b expression and degranulation

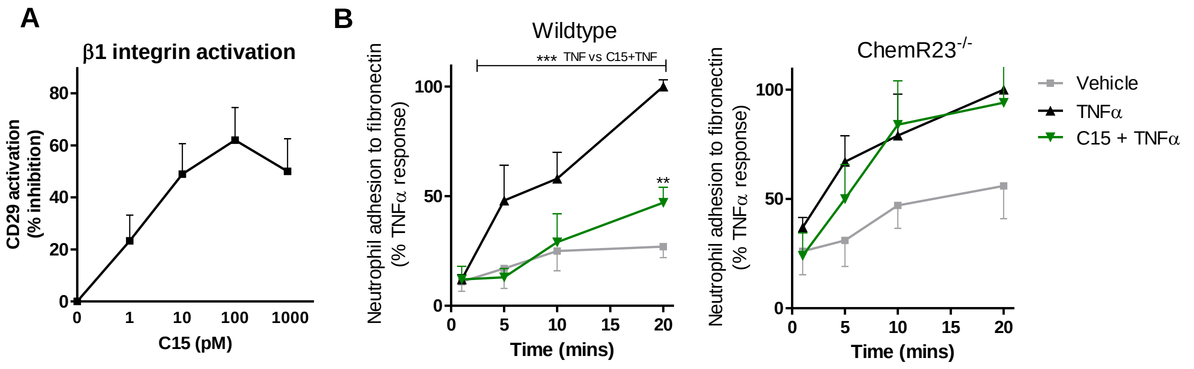

Supplementary Fig 3.

Effect of C15 on human neutrophil β1 integrin activation and adhesion of murine neutrophils to β1 ligand fibronectin

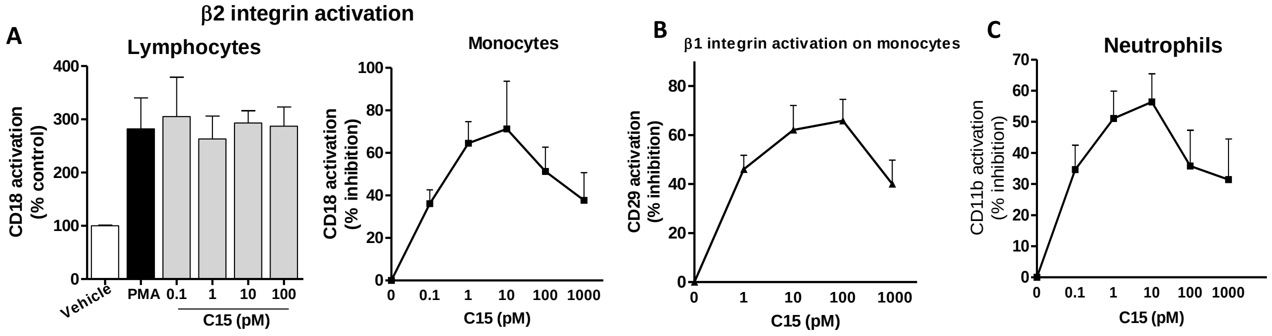

Supplementary Fig 4.
Specificity of C15's effects on integrin activation

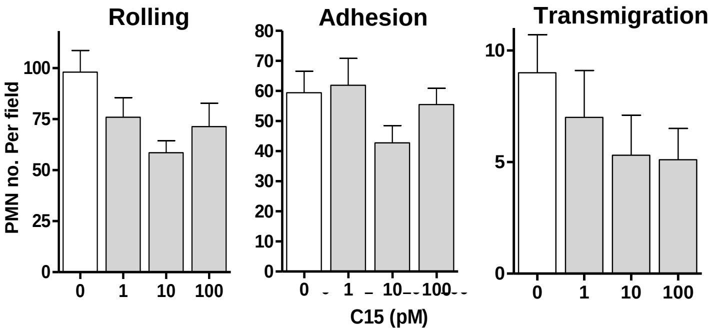

Supplementary Fig 5.

HUVEC treatment with C15 does not significantly affect neutrophil interactions
