# Supplementary figures and images for: Chemerin15 inhibits neutrophil-mediated vascular inflammation and myocardial ischemia-reperfusion injury through ChemR23
Source: EMBO Rep. 2013 Sep 3;14(11):999–1007. doi: 10.1038/embor.2013.138 (PMC3818079; doi:10.1038/embor.2013.138)

Fig.2C

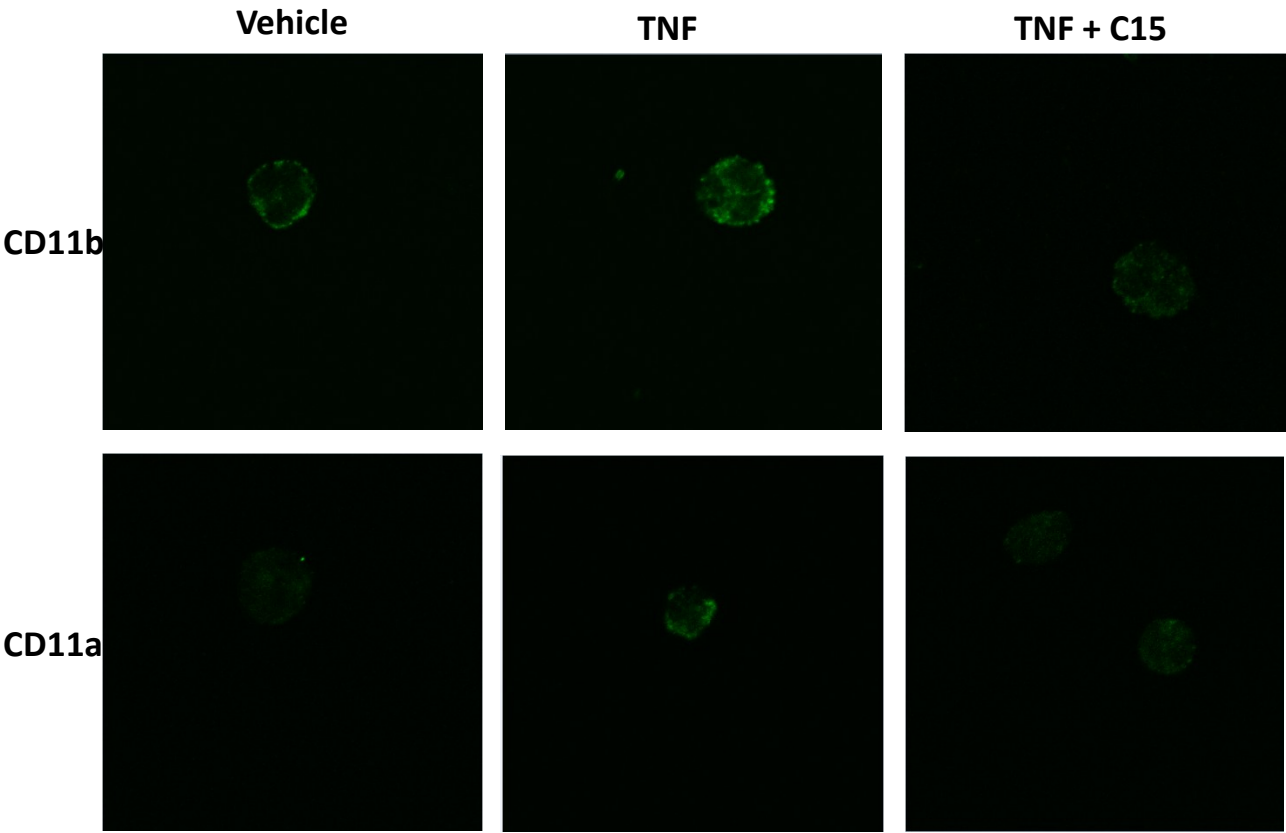

**Fig.2F**

**Vehicle**

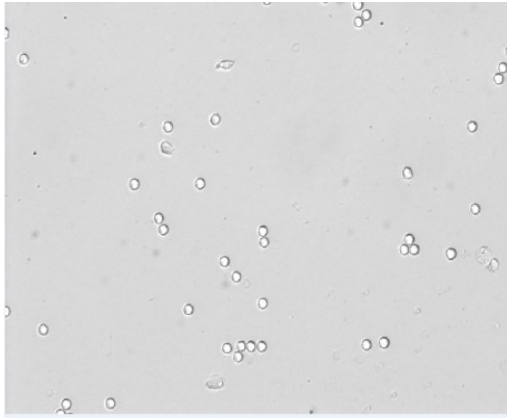

**TNF**

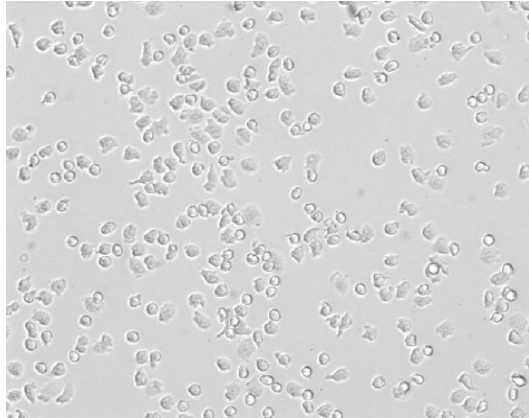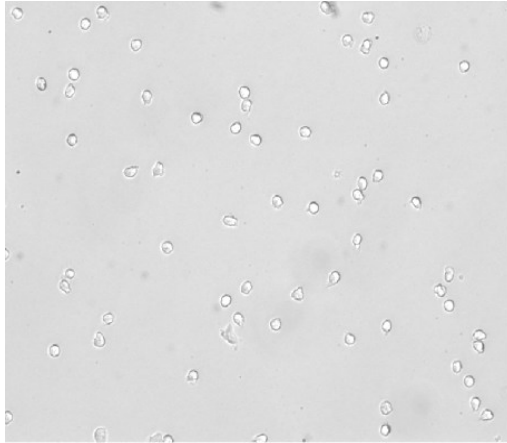

**TNF + C15**

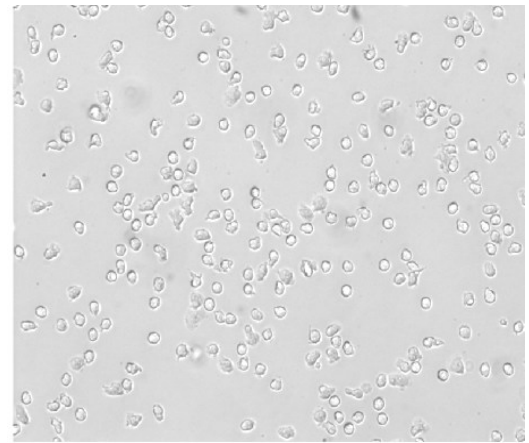

**TNF + C15 + CCX2005**

Fig.2I

Donor 1

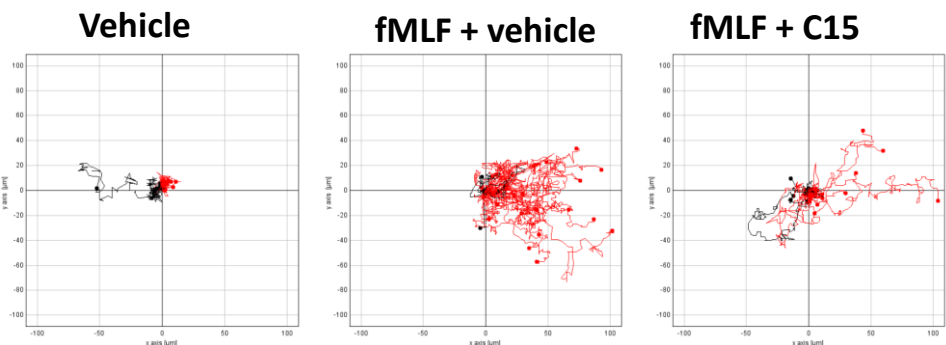

Donor 2

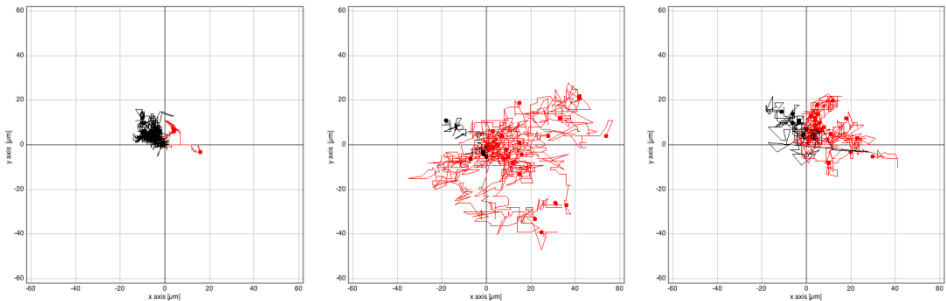

Donor 3

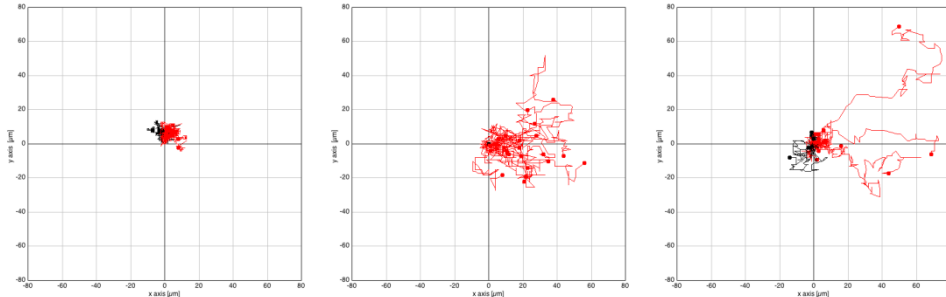

Donor 4

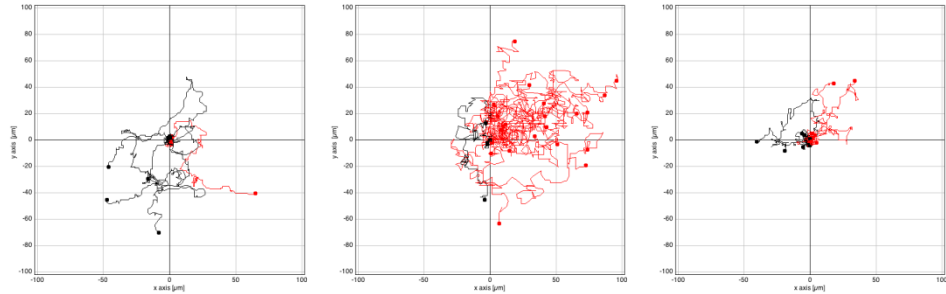

Donor 5

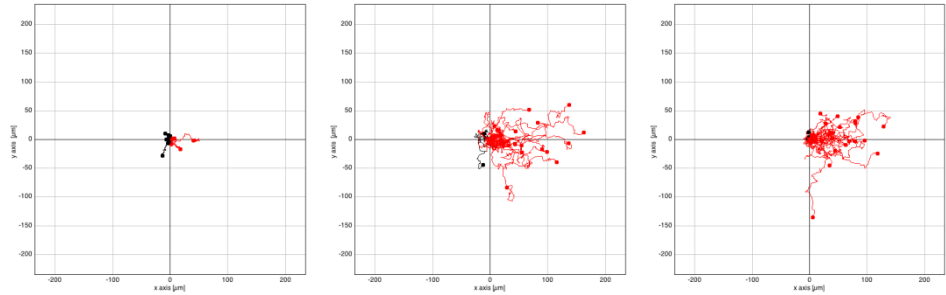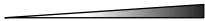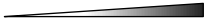

Supplement: Source Data for Figure 2 [file embor2013138df2a.pdf]
